# The first native Pyrgodesmidae (Diplopoda, Polydesmida) from Australia

**DOI:** 10.3897/zookeys.217.3809

**Published:** 2012-08-28

**Authors:** Robert Mesibov

**Affiliations:** 1 Queen Victoria Museum and Art Gallery, Launceston, Tasmania 7250, Australia

**Keywords:** Diplopoda, Polydesmida, Pyrgodesmidae, millipede, Australia, Queensland

## Abstract

Three new genera and six new species of Pyrgodesmidae are described from Queensland: *Asticopyrgodesmus* **gen. n.**, containing *Asticopyrgodesmus lamingtonensis* **sp. n.** and *Asticopyrgodesmus maiala* **sp. n.** (type species); *Nephopyrgodesmus* **gen. n.**, with *Nephopyrgodesmus eungella* **sp. n.** (type and only species); and *Notopyrgodesmus* **gen. n.**, with *Notopyrgodesmus kulla* **sp. n.** (type species), *Notopyrgodesmus lanosus* **sp. n.** and *Notopyrgodesmus weiri* **sp. n.** Localities and specimen data are given in an Appendix for undescribed Australian Pyrgodesmidae occurring in wet forests from the Northern Territory south to New South Wales, and on Lord Howe Island.

## Introduction

Pyrgodesmidae were first reported from Australia by Black (1997), who listed ‘Haplodesmidae and Pyrgodesmidae’ in his table of millipede family occurrences in Australian regions. The identification seemed a little uncertain: “Members of the families Haplodesmidae and Pyrgodesmidae are difficult to distinguish from one another. They are small, highly sculptured and rigid-bodied animals, widespread in soil and litter” (Black 1997, p. 558).

Together with haplodesmids and pyrgodesmids, Black may also have been recording superficially similar species of *Asphalidesmus* Silvestri, 1910 (Dalodesmidea), which are locally common in eastern Australia from northern Queensland to Tasmania (Mesibov 2011). The identity and relationships of *Asphalidesmus* were unclear when Black prepared his overview of the Australian millipede fauna.

Burrows et al. (1994) mentioned that a semi-aquatic polydesmidan, tentatively identified as a pyrgodesmid, had been collected on the campus of Macquarie University in Sydney, New South Wales in the early 1990s. The specimens were later identified as *Aporodesminus wallacei* Silvestri, 1904 (Pyrgodesmidae) by Adis et al. (1998), who published a redescription of this species. *Asticopyrgodesmus wallacei* has also been reported from St Helena (southern Atlantic Ocean), Tahiti and the Hawaiian Islands (Adis et al. 1998). It is a good example of a cryptogenic species (Carlton 1996), as it is not known to be native anywhere in its range.

**Figure 1.**
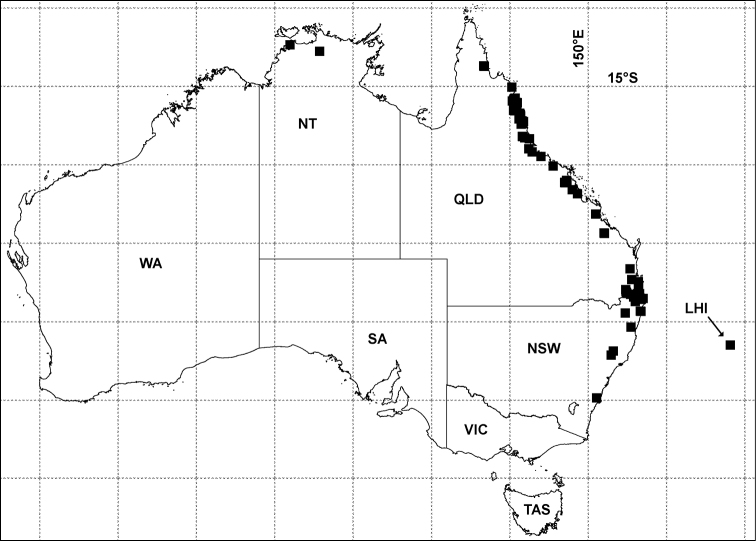
Localities for Pyrgodesmidae in Australia (black squares) as of July 2012. Geographic projection, 5° latitude-longitude grid. LHI = Lord Howe Island, NSW = New South Wales, NT = Northern Territory, QLD = Queensland, SA = South Australia, TAS = Tasmania, VIC = Victoria, WA = Western Australia. See Appendix for details of records.

As recently as 2011, Pyrgodesmidae were thought to be “unknown from Australia” (Shelley 2011, p. 7). Native pyrgodesmids are, in fact, common and readily collected in the wet forests of Queensland, and Australian museums hold specimens of Pyrgodesmidae from ca 14°S in northern Queensland to ca 35°S in southern New South Wales, a north-south range of ca 2300 km (Fig. 1; see Appendix for details of records). There are also Australian museum holdings of Pyrgodesmidae from the Northern Territory and Lord Howe Island (Fig. 1).

In this paper I sample the large diversity of native Australian pyrgodesmids by describing six new species from widely separated Queensland localities. I have redeposited as ‘Pyrgodesmidae’ another ca 400 specimens, representing an unknown number of additional species, in their respective Australian repositories (see Appendix). See the Discussion section for remarks on the taxonomic position of the three new genera described below.

## Methods

‘Male’ and ‘female’ in the text refer to adult individuals. All specimens are stored in 75–80% ethanol in their respective repositories.

Gonopods were cleared in 80% lactic acid and temporarily mounted in 60% lactic acid for optical microscopy, while other body parts were temporarily mounted in a 1:1 glycerol:water mixture. Preliminary drawings were made from views at 160X on a binocular microscope. Photomicrographs were taken with a Canon EOS 1000D digital SLR camera mounted on a Nikon SMZ800 binocular dissecting microscope equipped with a beam splitter. Measurements were made with the same microscope using an eyepiece scale. Some of the photomicrographs in this paper are composites, generated by focus-stacking with Zerene Stacker 1.04 software. Specimens for scanning electron microscopy were briefly air-dried, temporarily fixed to a stub with double-sided adhesive tape, examined uncoated with a FEI Quanta 600 operated in low-vacuum mode, then returned to alcohol. Images and drawings were prepared for publication using GIMP 2.6. Maps were generated using ArcView GIS 3.2.

Hoffman (1976) proposed a system for naming some commonly observed features of pyrgodesmid sculpturing. From Hoffman’s system I have taken the terms ‘paramedian tubercles’ and ‘dorsolateral tubercles’; see the text below and the figure captions for details. In describing details of microsculpture I use the terminology of Akkari and Enghoff (2011b).

The Appendix contains specimen data for the museum lots of Australian Pyrgodesmidae I examined. Locality details there and in the text below are given in all cases with latitude and longitude based on the WGS84 datum. The uncertainty for each locality is the radius of a circle around the stated position, in metres or kilometres.

Abbreviations: ANIC = Australian National Insect Collection, Canberra, Australian Capital Territory; Qld = Queensland; QM = Queensland Museum, Brisbane, Qld.

## Results

### Order Polydesmida Pocock, 1887. Suborder Polydesmidea Pocock, 1887. Family Pyrgodesmidae Silvestri, 1896

#### Genus Asticopyrgodesmus

Mesibov gen. n.

urn:lsid:zoobank.org:act:736D393F-70B0-43F6-9A99-F7771E877984

http://species-id.net/wiki/Asticopyrgodesmus

##### Type species.

*Asticopyrgodesmus maiala* Mesibov, sp. n., by present designation.

##### Other assigned species.

*Asticopyrgodesmus lamingtonensis* Mesibov, sp. n.

##### Diagnosis.

With head + 19 or head + 20 rings; collum not covering head in dorsal view; metatergal tubercles patterned in 4 transverse rows; no enlargement of paramedian or dorsolateral tubercles; ozopores on porosteles; gonopod telopodite distally with rod-like, medial solenomere curving laterally towards much larger, mediolaterally flattened lateral branch.

##### Etymology.

Greek *astikos*, ‘urban’ (referring to the occurrence of the type species in the most densely populated part of Queensland), + the type genus of the family, *Pyrgodesmus* Pocock, 1892; gender masculine.

#### Asticopyrgodesmus maiala

Mesibov sp. n.

urn:lsid:zoobank.org:act:0A82744F-470C-4E28-9F49-774229BC8FF4

http://species-id.net/wiki/Asticopyrgodesmus_maiala

**Figure 2.**
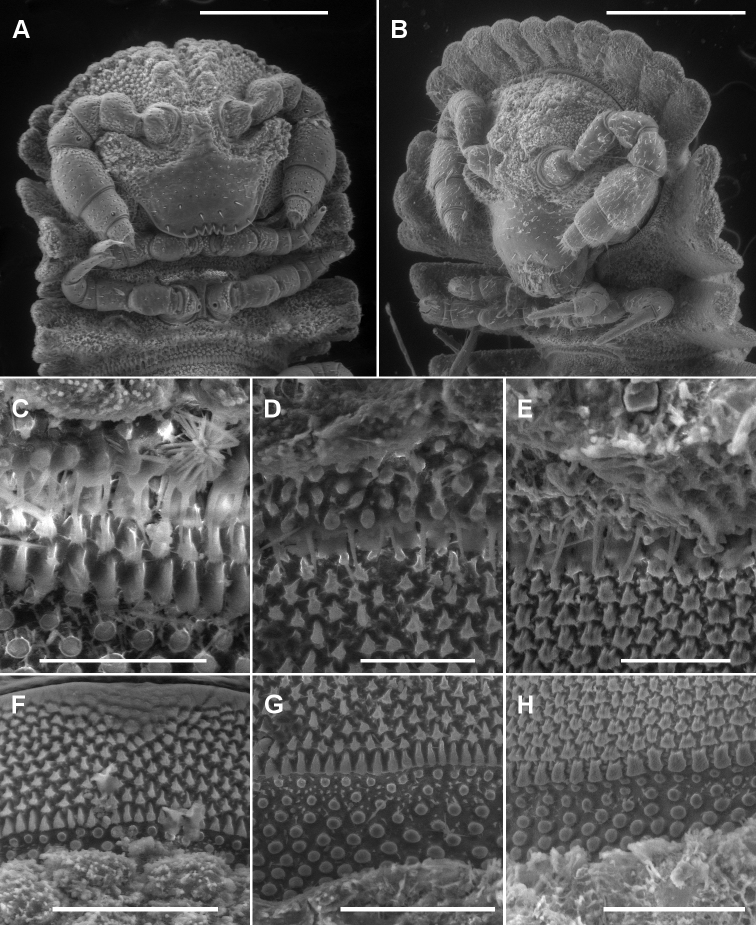
**A, B** Ventral views of head of *Asticopyrgodesmus maiala* sp. n., male paratype ex ANIC 64-000220 (**A**) and *Notopyrgodesmus weiri* sp. n., male paratype ANIC 64-000249 **C, D, E** Views of lobe-and-spike limbus on midbody rings (anterior at top) of *Asticopyrgodesmus maiala* sp. n., male paratype ex ANIC 64-000220 (**C**), *Nephopyrgodesmus eungella* sp. n., male paratype ex ANIC 64-000231 (**D**) and *Notopyrgodesmus kulla* sp. n., male paratype ex ANIC 64-000243 (**E**) **F, G, H** Views of prozonite sculpture on midbody rings (anterior at top) of *Asticopyrgodesmus lamingtonensis* sp. n., male paratype ex ANIC 64-000217 (**F**), *Notopyrgodesmus eungella* sp. n., male paratype ex ANIC 64-000231 (**G**) and *Notopyrgodesmus kulla* sp. n., male paratype ex ANIC 64-000243 (**H**). Scanning electron micrographs of uncoated specimens; scale bars: **A** = 0.5 mm, **B** = 0.2 mm, **C**, **D**, **E, H** = 0.05 mm, **F**, **G** = 0.1 mm.

**Figure 3.**
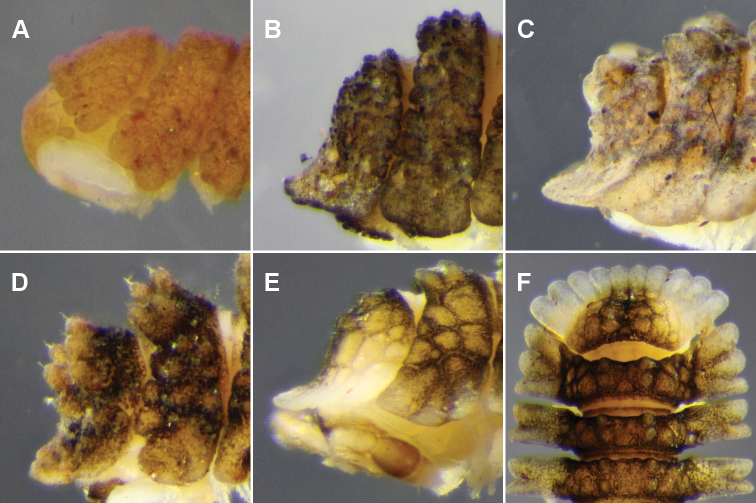
**A–E** Left lateral views of anterior end. **A** *Asticopyrgodesmus maiala* sp. n., holotype **B** *Nephopyrgodesmus eungella* sp. n., male paratype ex ANIC 64-000231 **C** *Notopyrgodesmus kulla* sp. n., male paratype ex ANIC 64-000242 **D** *Nephopyrgodesmus lanosus* sp. n., holotype **E** *Nephopyrgodesmus weiri* sp. n., holotype **F** *Nephopyrgodesmus weiri* sp. n., male paratype ANIC 64-000251, dorsal view of anterior end. Images not to same scale.

**Figure 4.**
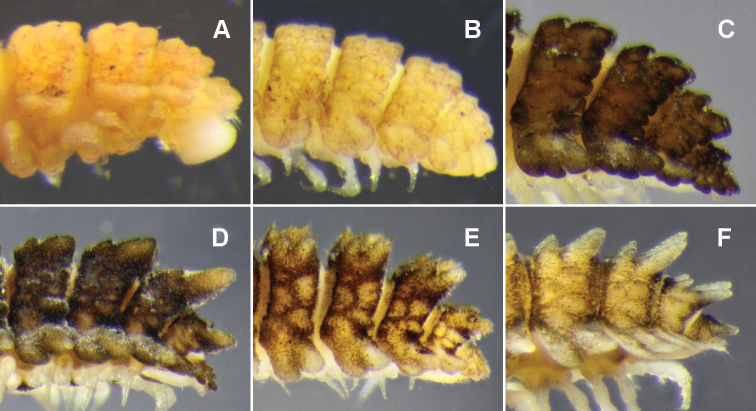
Left lateral views of posterior end of holotype. **A** *Asticopyrgodesmus maiala* sp. n. **B** *Asticopyrgodesmus lamingtonensis* sp. n. **C** *Nephopyrgodesmus eungella* sp. n. **D** *Notopyrgodesmus kulla* sp. n. **E** *Notopyrgodesmus lanosus* sp. n. **F** *Notopyrgodesmus weiri* sp. n. Images not to same scale.

**Figure 5.**
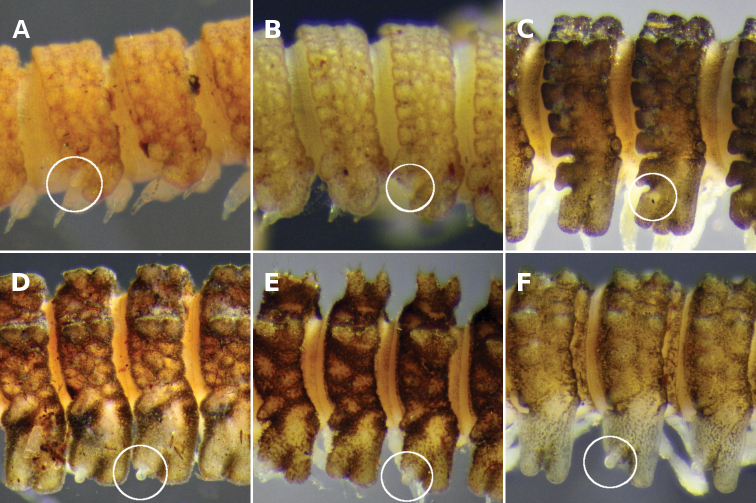
Right dorsolateral views of midbody, anterior to right. **A** *Asticopyrgodesmus maiala* sp. n., holotype **B** *Asticopyrgodesmus lamingtonensis* sp. n., holotype **C** *Nephopyrgodesmus eungella* sp. n., holotype **D** *Notopyrgodesmus kulla* sp. n., male paratype ex ANIC 64-000242 **E** *Notopyrgodesmus lanosus* sp. n., holotype **F** *Notopyrgodesmus weiri* sp. n., holotype. Images not to same scale. White circles: ozopore or porostele.

**Figure 6.**
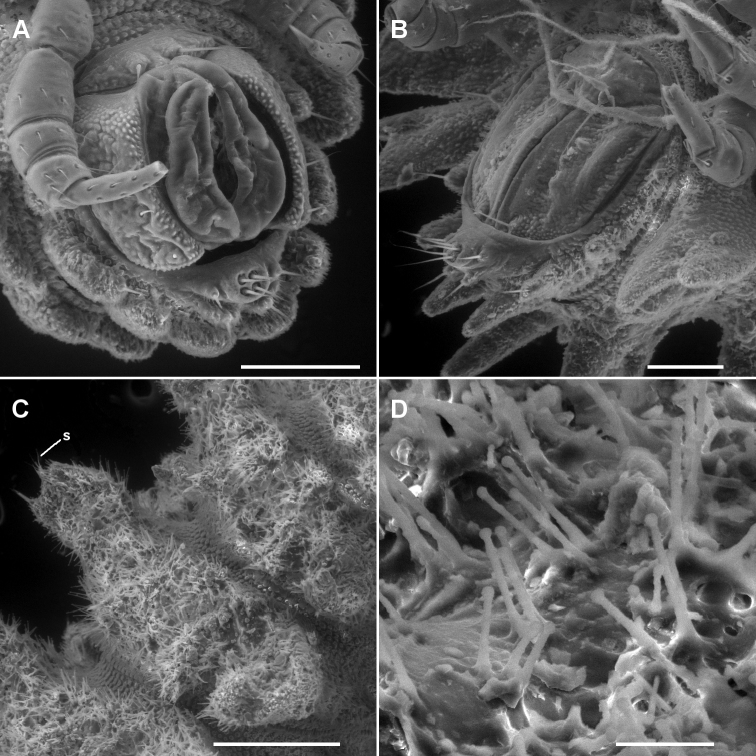
**A, B** Ventrolateral views of telson of *Asticopyrgodesmus maiala* sp. n., male paratype ex ANIC 64-000220 (**A**) and *Notopyrgodesmus weiri* sp. n., male paratype ANIC 64-000249 (**B**) **C** Right lateral view of midbody ring of *Notopyrgodesmus kulla* sp. n., male paratype ex ANIC 64-000243, anterior to upper right; **s** = ‘spine’ on tip of paramedian tubercle **D** Close-up of hair-like cuticular outgrowths on specimen in **C** Scanning electron micrographs of uncoated specimens; scale bars: **A**, **B** = 0.1 mm, **C** = 0.25 mm, **D** = 0.025 mm.

**Figure 7.**
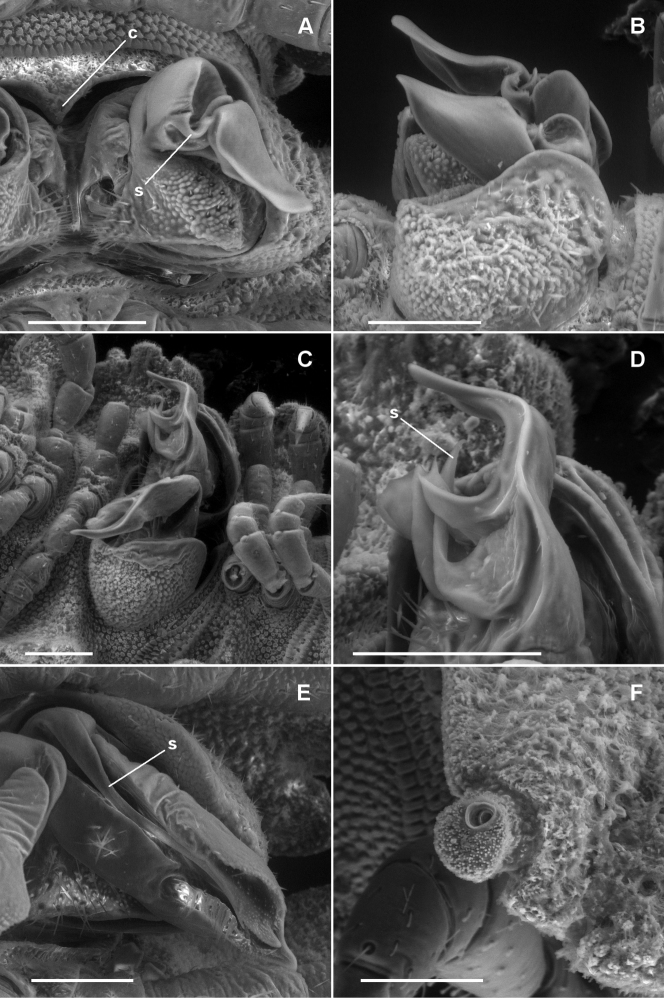
**A**, **B** *Asticopyrgodesmus maiala* sp. n., male paratypes ex ANIC 64-000220. **A** Ventral view (anterior at top) of left gonopod; **c** = aperture constriction, **s** = solenomere **B** Left lateral view of gonopods in situ **C**, **D** *Asticopyrgodesmus lamingtonensis* sp. n., male paratype ex ANIC 64-000217 **C** Left ventrolateral view of gonopods in situ **D** Close-up of view in **C**; **s** = solenomere. (See also Fig. 8A) **E** *Nephopyrgodesmus eungella* sp. n., male paratype ex ANIC 64-000231, ventral view of left gonopod telopodite; **s** = solenomere (see also Fig. 8B) **F** *Notopyrgodesmus kulla* sp. n., male paratype ex ANIC 64-000243, left lateral view of midbody porostele. Scanning electron micrographs of uncoated specimens; scale bars = 0.1 mm.

Figs 2A, 2C , 3A , 4A , 5A , 6A , 7A, 7B

##### Holotype.

Male, Maiala National Park, Qld, 27°20'S, 152°46'E ±2 km, 635 m, 13 March 1973, R.J. Kohout, ANIC berlesate 450, rainforest, ANIC 64-000219.

**Paratypes.** **ANIC:** 7 males, 1 female, details as for holotype, 64-000218; 9 males, 7 females, same details but R.W. Taylor, ANIC berlesate 451, 64-000220.

##### Other material.

**ANIC:** 38 males, 4 females, 1 stadium 6 female, Joalah National Park [now the Joalah section of Tamborine National Park], Qld, 27°55'S, 153°12'E ±2 km, 380 m, 14 March 1973, R.J. Kohout, ANIC berlesate 454, rainforest, 64-000221; 13 males, 14 females, same details but ANIC berlesate 453, 64-000222; 8 males, same details but ANIC berlesate 452, 64-000223; 9 males, same locality but 27°56'S, 153°12'E ±2 km, 18-21 October 1978, J. Lawrence and T.A. Weir, ANIC berlesate 653, rainforest litter, 64-000224; 10 males, 2 females, same locality but 27°58'S, 153°11'E ±2 km, 23 July 1979, J. Lawrence, ANIC berlesate 656, flood debris and leaf litter, 64-000225; 2 males, O'Reillys, Lamington National Park, Qld, 28°14'S, 153°08'E ±2 km, 920 m, 21 March 1973, R.W. Taylor, ANIC berlesate 459, rainforest, 64-000226; 14 males, 1 female, same locality but 22-27 November 1978, J. Lawrence and T.A. Weir, ANIC berlesate 655, rainforest litter, 64-000227. **QM:** 5 males, 6 females, O'Reillys Guesthouse, Lamington National Park, Qld, 28°14'08"S, 153°08'12"E ±500 m, 900 m, 14 December 1981, R. Raven, G. Monteith and D. Yeates, QM berlesate 377, rainforest, sieved litter, S92790; 1 male, Mistake Mountains via Goomburra, Sylvesters Track, Qld, 27°58'37"S, 152°22'58"E ±500 m, 1040 m, 21-22 November 1987, P.M. Johns, rainforest logs and litter, S92788; 1 male, Springbrook Repeater, Qld, 28°14'23"S, 153°15'58"E ±500 m, 1000 m, 14 March 1997, G. Monteith and E. Russell, QM berlesate 922, rainforest, sieved litter, S40086; 2 males, 1 female, Mt Superbus, Qld, 28°13'31"S, 152°27'17"E ±500 m, 1370 m, 24 October 1998, G. Monteith, QM berlesate 973, rainforest, stick brushing, S92789.

**Diagnosis.** Readily distinguished from *Asticopyrgodesmus lamingtonensis* sp. n. in having head + 19 rings, anterior margin of paranota higher than posterior margin, ozopores on porosteles on rings 5, 7, 9, 10, 12, 13, 15.

##### Description.

Colour in alcohol light yellowish brown with sparse encrustation of soil particles on vertex, collum, tergites, metatergites and preanal ring. Males and females with head + 19 rings. Male and female approximate measurements: length ca 5 mm; ring 12: overall width 0.7 mm, overall width/prozonite width 1.5, maximum vertical diameter 0.5 mm.

Head (Fig. 2A) with vertex and frons roughened; largest tubercles in rows either side of vertigial sulcus; clypeus smooth and setose. Postantennal groove deep; antennal sockets separated by ca 1× a socket diameter; antennomere relative widths 5>6>(2,3,4), relative lengths 5>2>(3,4,6).

Collum not covering head in dorsal view (Fig. 3A); anterior margin divided into 10 rounded lobes. Overall ring widths 2–14 about equal, gradually decreasing on rings 15–18.

Midbody metatergite sculpture usually obscured by encrusted soil particles, patterned in 4 transverse rows of variably sized, rounded tubercles; enlarged paramedian or dorsolateral tubercles not evident on any rings (Fig. 4A). Anterior metatergal margins indistinctly divided into ca 16 lobes, posterior margin into 14 lobes, the lateralmost lobe on each side largest and most clearly demarcated.

Paranota (Fig. 5A) very short, arising low on body, slightly declined, pitched so anterior margin is higher than posterior margin. Ring 2 paranotum expanded anterodistally, lateral margin weakly divided into 3 rounded lobes. From ring 3 onwards, anterior margin very slightly convex, undivided; 2 weakly demarcated lateral marginal lobes, the anterior lobe larger; posterior margin short, slightly convex.

Metatergal surface details obscured by soil particles and secretions; no setae evident on collum, tergites or metatergites. Prozonites with lozenge-shaped raised elements anterior to suture and subspherical knobs posterior to suture; suture marked by a ridge of longitudinally elongated elements. Limbus a single row of very thin lobes with more or less straight distal margins, and with 1–2 very thin spikes above each lobe (Fig. 2C).

Ozopores (Fig. 5A) on prominent porosteles arising from posterobasal corner of paranota on rings 5, 7, 9, 10, 12, 13, 15.

Sternites not setose, about as wide as long; transverse impression wider than longitudinal. Legs short, hidden by paranota in dorsal view; relative podomere lengths tarsus>femur>prefemur>(postfemur, tibia). Spiracles not evident.

Preanal ring (Fig. 6A) with 4+4 lobes; epiproct very short, bifurcate; spinnerets in square array below apex; hypoproct rounded-triangular.

Gonopore a small, inconspicuous opening on medial surface of leg 2 coxa (Fig. 2A). Aperture transversely rectangular, ca 1/2 width of ring 7 prozonite, slightly constricted anteriorly at midline (Fig. 7A). Gonocoxa covering most of telopodite with roughened, sparsely setose, slightly convex outer surface when gonopods retracted. Gonocoxa deeply excavate medially, forming gonocoel. Telopodite (Fig. 7A) with wide base in gonocoel; tapering abruptly from base, swollen and roughened posteroventrally, sparsely setose, the posteromedial corner of the base extended and more densely setose. Distal portion of telopodite (Figs 7A, B) divided into medial and lateral branches; medial branch cup-like, the posteromedial rim of the cup produced as short, distolaterally curving solenomere; lateral branch of telopodite extending posterolaterally as tongue-shaped process, slightly concave ventrolaterally. Prostatic groove running on medial surface of medial branch of telopodite, opening at tip of solenomere.

Female with epigyne ca 1/2 ring width, slightly raised, rounded-rectangular with straight distal margin; cyphopods not examined.

##### Distribution.

Rainforest in southeastern Queensland, from D’Aguilar National Park northwest of Brisbane almost to the New South Wales border, a north-south distance of ca 110 km (Fig. 11). Co-occurs with *Asticopyrgodesmus lamingtonensis* sp. n. in Lamington National Park.

##### Etymology.

For the Maiala section of D’Aguilar National Park, the type locality; noun in apposition.

##### Remarks.

Two of the males in ANIC 64-000220 are noticeably smaller than the other seven in the sample, but seem to have the same gonopod structure in situ at high optical magnification. While the smaller ‘*Asticopyrgodesmus maiala*’ specimens may represent a third species of *Asticopyrgodesmus*, I have not been able to detect differences in somatic features between them and the seven larger males in ANIC 64-000220.

#### Asticopyrgodesmus lamingtonensis

Mesibov sp. n.

urn:lsid:zoobank.org:act:F82C6EA1-C46A-42CA-8E79-5AAA254065CC

http://species-id.net/wiki/Asticopyrgodesmus_lamingtonensis

**Figure 8.**
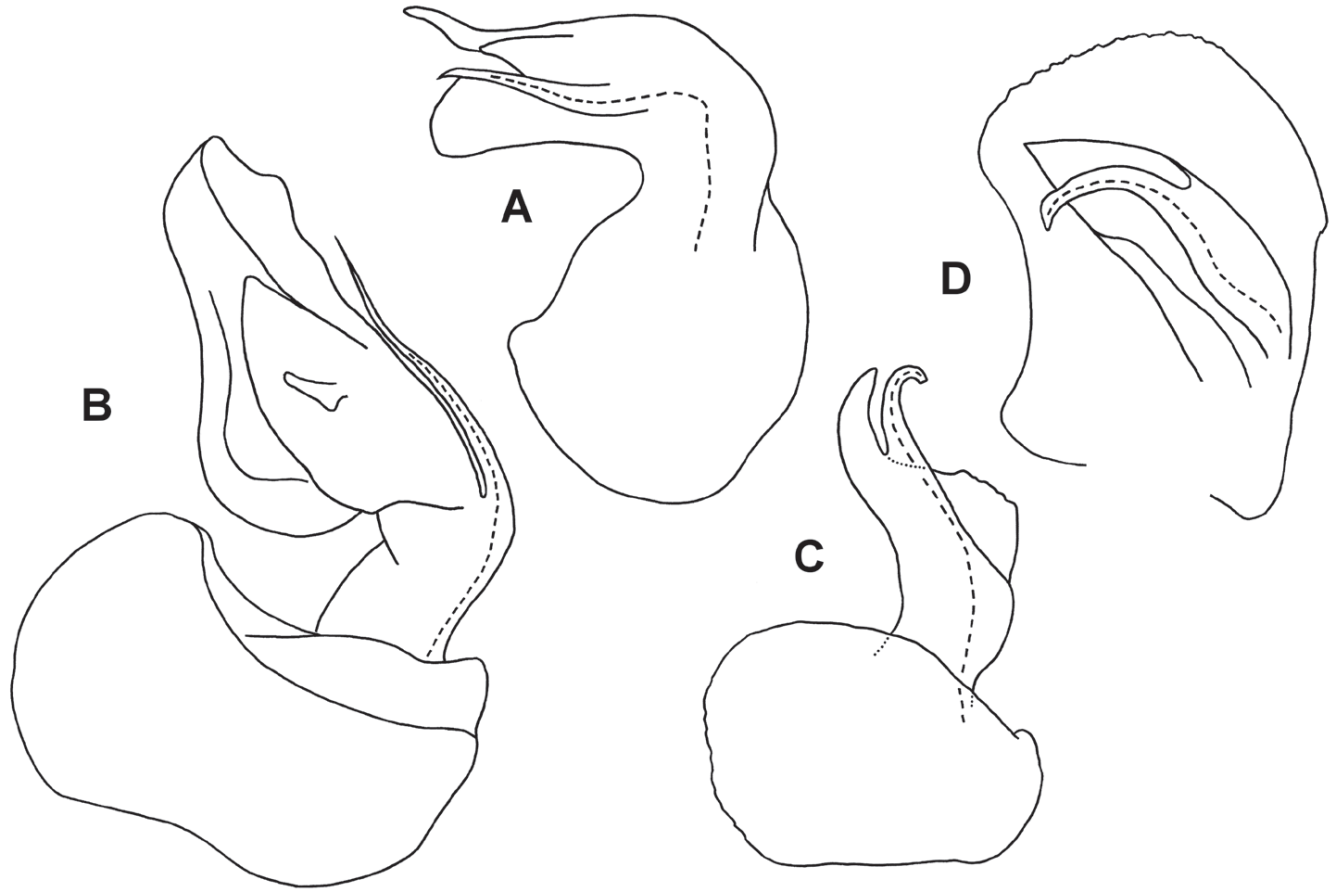
**A** Medial view of right gonopod telopodite of *Asticopyrgodesmus lamingtonensis* sp. n., paratype ex ANIC 64-000217 (see also Figs 7C, 7D) **B** Posterior view of right gonopod of *Nephopyrgodesmus eungella* sp. n., paratype ex ANIC 64-000231 (see also Fig. 7E) **C** Anterior view of left gonopod of *Notopyrgodesmus kulla* sp. n., QM S92795 **D** Medial view of right gonopod (retracted) of *Notopyrgodesmus kulla* sp. n., paratype ex ANIC 64-000239. Setation not shown; dashed line indicates course of prostatic groove. Drawings not to same scale.

Figs 2F , 4B , 5B , 7C, 7D , 8A

##### Holotype.

Male, O’Reillys, Lamington National Park, Qld, 28°14'S, 153°08'E ±2 km, 22–27 November 1978, J. Lawrence and T. Weir, ANIC berlesate 655, rainforest litter, ANIC 64-000216.

##### Paratypes.

**ANIC:** 12 males, details as for holotype, 64-000217.

##### Other material.

None.

##### Diagnostic description.

As for *Asticopyrgodesmus maiala* sp. n., differing in the following details (see also Figs 2F, 4B):

Males with head + 20 rings (females not yet recognised). Paranota (Fig. 5B) extending further laterally than in *Asticopyrgodesmus maiala* sp. n., anterior edge lower than posterior edge, declined at ca 60°. Ozopores on porosteles on rings 5, 7, 9, 12, 15 only. Gonopod telopodite (Figs 7C, 7D, 8A) divided distally into three branches: (a) basal branch posteriorly directed, mediolaterally flattened, apically with broad, gently convex margin; (b) solenomere rod-like, medial, tapering to point, posteriorly directed at base and abruptly bent posterolaterally; (c) laterodistal branch directed posterolaterally, subdivided into two mediolaterally flattened processes directed posteriorly with wide, U-shaped gap between, the lateral process larger, both processes tapering to rounded apex.

##### Distribution.

So far known only from rainforest at the type locality in southeastern Queensland (Fig. 11). Co-occurs with *Asticopyrgodesmus maiala* sp. n.

##### Etymology.

For Lamington National Park, the type locality; adjective.

##### Remarks.

This species very closely resembles *Asticopyrgodesmus maiala* sp. n., but differs most obviously in ring number.

#### Genus Nephopyrgodesmus

Mesibov gen. n.

urn:lsid:zoobank.org:act:3C60EF58-732B-4429-9DCF-46567E167E06

http://species-id.net/wiki/Nephopyrgodesmus

##### Type and only species.

*Nephopyrgodesmus eungella* Mesibov, sp. n., by present designation.

##### Diagnosis.

Males and females with head + 20 rings; collum completely covering head in dorsal view; metatergal tubercles patterned in 3 transverse rows; paramedian and dorsolateral tubercles slightly enlarged; ozopores not on porosteles; gonopod telopodite divided distally into medial, flagellum-like solenomere and much larger lateral branch, the latter divided into appressed medial and lateral processes, with the solenomere normally lying parallel to and just above the line of contact between the two processes.

##### Etymology.

Greek *nephos*, ‘cloud’ (referring to the place name Eungella, reported to mean ‘land of cloud’), + the type genus of the family, *Pyrgodesmus*; gender masculine.

#### Nephopyrgodesmus eungella

Mesibov sp. n.

urn:lsid:zoobank.org:act:B3FF17F4-C15C-400C-9329-C628588393E0

http://species-id.net/wiki/Nephopyrgodesmus_eungella

**Figure 9.**
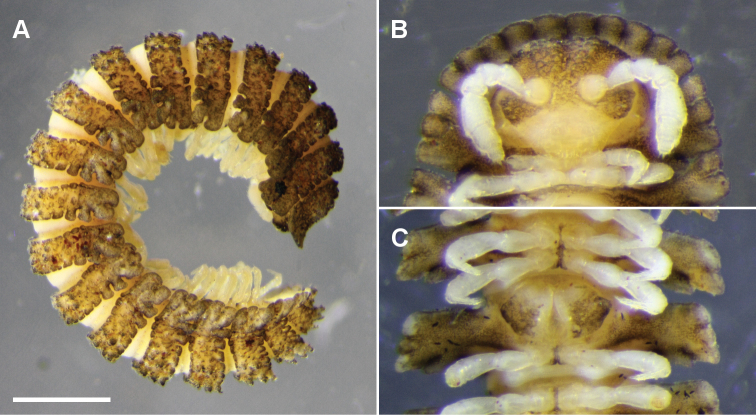
*Nephopyrgodesmus eungella* sp. n., male paratypes ex ANIC 64-000228. **A** Whole animal, left lateral view; scale bar = 1 mm **B** Anterior end, ventral view **C** Retracted gonopods, ventral view **B, C** not to same scale.

Figs 2D , 3B , 4C , 5C , 9A–C , 7E , 8B

##### Holotype.

Male, Eungella National Park, Qld, 21°09'S, 148°30'E ±2 km, 760 m, 10 November 1976, R.W. Taylor and T.A. Weir, ANIC berlesate 562, rainforest, ANIC 64-000232.

##### Paratypes.

**ANIC:** 9 males, 13 females, details as for holotype, 64-000231; 6 males, 6 females, same details but ANIC berlesate 563, 64-000229; 10 males, 9 females, same details but ANIC berlesate 564, 64-000228; 1 male, 2 females, Broken River, Eungella National Park, Qld, 21°10'S, 148°31'E ±2 km, 700 m, 10-12 November 1976, R.W. Taylor and T.A. Weir, ANIC berlesate 559, 64-000234; 3 males, 1 female, same details but ANIC berlesate 560, 64-000237; 4 males, 3 females, same details but ANIC berlesate 561, 64-000235; 5 males, 2 females, same details but ANIC berlesate 568, 64-000236; 4 males, 2 females, same details but ANIC berlesate 570, 64-000233.

##### Other material.

**ANIC:** 3 males, 3 females, 3 stadium 7 females, 3 km S of Eungella, Qld, 21°09'S, 148°29'E ±2 km, 780 m, 26 March 1975, R.W. Taylor, ANIC berlesate 489, 64-000230; 4 males, 5 females, 1 stadium 6 female, Finch Hatton Gorge, Qld, 21°05'S, 148°38'E ±2 km, 200 m, 11 November 1976, R.W. Taylor and T.A. Weir, ANIC berlesate 565, rainforest, 64-000238. **QM:** 1 female, Eungella schoolhouse, Qld, 21°07'51"S, 148°29'32"E ±2 km, 13 February 1986, J. Gallon and R. Raven, QM berlesate 709, rainforest, S92792; 4 females, Finch Hatton Gorge, Qld, 21°04'13"S, 148°38'11"E ±500 m, 300 m, 18 November 1992, G. Monteith, G. Thompson, D. Cook and H. Janetzki, S92791.

##### Description.

Colour in alcohol pale yellow, with brown encrusted soil particles on vertex, frons, collum, tergites, metatergites, preanal ring, gonocoxae and midline of sternites (Fig. 9A). Males and females with head + 20 rings. Male and female approximate measurements: length 7 mm; ring 12: overall width 1.5 mm, overall width/prozonite width 2.2, maximum vertical diameter 0.8 mm.

Head (Fig. 9B) with vertex and frons roughened, clypeus smooth and setose. Postantennal groove deep; antennal sockets separated by ca 1× a socket diameter; antennomere relative widths 5>6>(2,3,4), relative lengths 5>(2,6)>(3,4).

Collum completely covering head in dorsal view (Fig. 3B); anterior margin of collum parallel to ground, weakly divided into 10 rounded lobes; main part of collum nearly perpendicular to ground in lateral view. Overall ring widths 2–17 about equal; rings 18 and 19 narrower.

Midbody metatergite sculpture usually obscured by encrusted soil particles, patterned in 3 transverse rows; 3 small, short, rounded-conical paramedian tubercles, the posterior pair sometimes confluent; 2-3 small, short, rounded-conical dorsolateral tubercles, the second tubercle largest. Paramedian tubercles only slightly enlarged on rings 17–19 (Fig. 4C). Anterior metatergal margin typically weakly divided into 14 rounded lobes; posterior metatergal margin into 12 subquadrate lobes.

Paranota (Fig. 5C) arising low on body, very slightly directed posteriorly, declined at ca 45°, pitched slightly so anterior margin is lower than posterior margin. Ring 2 paranotum expanded anterodistally, lateral margin very weakly divided into 3 rounded lobes. From ring 3 onwards, anterior margin straight, undivided; 3 weakly demarcated lateral marginal lobes on poriferous rings, 2 on non-poriferous rings; posterior margin with deep notch dividing margin into 2 lobes with straight distal margins, the basal lobe smaller. Rings 18 and 19 paranota directed posterolaterally.

Metatergal surface details obscured by soil particles and secretions; no setae evident on collum, tergites or metatergites. Prozonites with lozenge-shaped raised elements anterior to suture and subspherical knobs posterior to suture; suture marked by a ridge of longitudinally elongated elements. Limbus a single row of very thin lobes with more or less straight distal margins, and with 1-2 very thin spikes above each lobe (Fig. 2D).

Ozopores (Fig. 5C) not on porosteles, difficult to detect, opening near medial, basal corner of largest (most lateral) posterior marginal lobe of paranota on rings 5, 7, 9, 10, 12, 13, 15–17 (and 18 and 19?).

Sternites slightly roughened, not setose, longer than wide; transverse impression deeper than longitudinal. Legs short, hidden by paranota in dorsal view; relative podomere lengths (femur, tarsus)>prefemur>(postfemur, tibia). Spiracles small; anterior spiracle above anterior leg, posterior spiracle above and anterior to posterior leg.

Preanal ring with 3+3 lobes; epiproct short, bluntly rounded, spinnerets in square array below apex; hypoproct rounded-triangular.

Gonopore a small, inconspicuous opening on medial surface of leg 2 coxa. Aperture tranversely ovoid, between 1/3 and 1/2 width of ring 7 prozonite. Gonocoxa shallowly concave medially, forming gonocoel, with a few setae on outer ventromedial and inner dorsomedial surfaces, and with small, setose knob on medial surface near base. Telopodite (Figs 7E, 8B) arising on anteromedial surface of gonocoel, cylindrical near base, the distal two-thirds bent posterolaterally and divided into a large, complex, main branch and a flagellum-like, anteromedial solenomere. Main branch subdivided into lateral and medial portions; lateral portion slightly flattened mediolaterally, posterior margin expanded basally, anterior margin folded over medially; medial portion of main branch ca 2/3 length of lateral portion and narrower, apex acuminate, closely pressed to lateral portion, and with small, thin tab on medial surface directed posteromedially. Solenomere a little longer than medial portion of main branch, normally lying in groove between folded-over anterior margin of lateral portion of main branch and anterior margin of medial portion (Fig. 7E). Prostatic groove running on anteromedial surface of telopodite base before entering flagellum, apparently opening on flagellum tip. When retracted (Fig. 9C), gonocoxae forming two low, rounded mounds, the distomedial edges almost meeting anteriorly but separated posteriorly, exposing anterior surface of telopodite.

Female with epigyne ca 1/2 ring width, slightly raised, rounded-rectangular with straight distal margin; cyphopods not examined.

##### Distribution.

Known from rainforest in the Eungella area in central (near-coastal) Queensland and from Finch Hatton Gorge, ca 15 km distant (Fig. 11).

##### Etymology.

For Eungella National Park, the type locality; noun in apposition. The name Eungella is “Derived from [the] town and pastoral run name first used by Ernest Favenc (1845-1908), explorer, journalist and historian, in July 1876, reportedly an Aboriginal word, language and dialect not recorded, indicating land of cloud” (http://www.derm.qld.gov.au/property/placenames/details.php?id=46868; accessed 12 July 2012).

##### Remarks.

*Nephopyrgodesmus eungella* is one of the few Australian pyrgodesmids which can be found partly coiled in preservation (Fig. 9A).

#### Genus Notopyrgodesmus

Mesibov gen. n.

urn:lsid:zoobank.org:act:7110AB73-F50B-449A-9B32-8531DFFA3B7E

http://species-id.net/wiki/Notopyrgodesmus

##### Type species:

*Notopyrgodesmus kulla* Mesibov, sp. n., by present designation.

##### Other assigned species.

*Notopyrgodesmus lanosus* Mesibov, sp. n., *Notopyrgodesmus weiri* Mesibov, sp. n.

##### Diagnosis.

Males and females with head + 20 rings; collum completely covering head in dorsal view; metatergal tubercles patterned in 2 transverse rows; paramedian and dorsolateral tubercles prominent, much enlarged on last rings; ozopores on porosteles; tergites and metatergites dorsally with minute, hair-like cuticular outgrowths with expanded tips; gonopod telopodite distally like curled leaf, concave medially, the rod-like solenomere arising from curled anteromedial margin of telopodite.

##### Etymology.

Greek *notos* (‘south’) + the type genus of the family, *Pyrgodesmus*; gender masculine.

#### Notopyrgodesmus kulla

Mesibov sp. n.

urn:lsid:zoobank.org:act:A3DAEF00-932C-443E-B8AB-7859A6DF460B

http://species-id.net/wiki/Notopyrgodesmus_kulla

**Figure 10.**
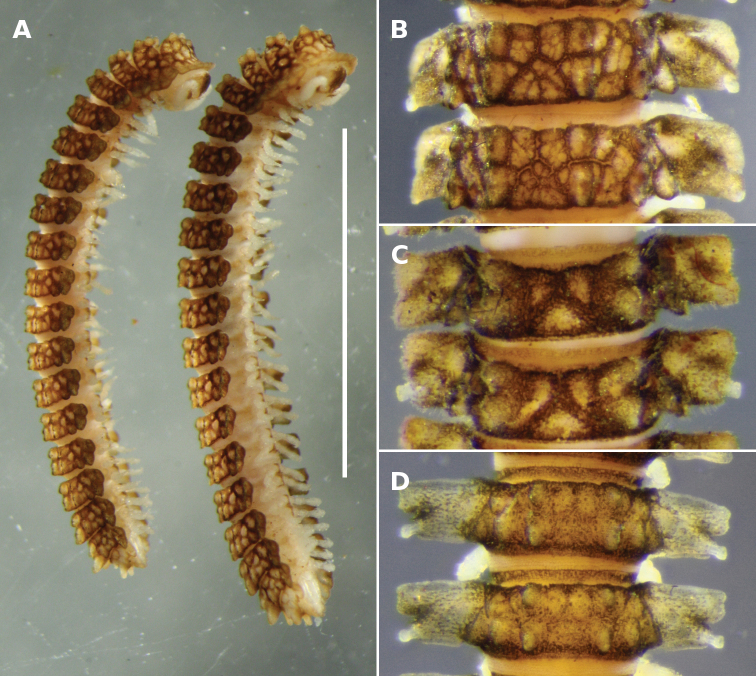
**A** *Notopyrgodesmus kulla* sp. n., male (left) and female (right) paratypes ex QM S92793. Scale bar = 5 mm **B–D** Dorsal views of midbody rings of males, not to same scale **B** *Notopyrgodesmus kulla* sp. n., paratype ex QM S92793 **C** *Notopyrgodesmus lanosus* sp. n., holotype **D** *Notopyrgodesmus weiri* sp. n., paratype ANIC 64-000251.

Figs 2E , 3C , 4D , 5D , 7F , 8C, 8D , 10A, 10B

##### Holotype.

Male, 11 km W by N of Bald Hill, McIlwraith Range, Qld, 13°44'S, 143°20'E ±2 km, 520 m, 27 June - 12 July 1989, T.A. Weir, ANIC berlesate 1113, search party campsite, closed forest, flood debris, ANIC 64-000240.

##### Paratypes.

**ANIC:** 3 males, 3 females, details as for holotype, 64-000239; 1 male, 2 females, same details but ANIC berlesate 1111, leaf and log litter, 64-000242; 1 female, 1 stadium 7 male, same details but ANIC berlesate 1114, leaf litter and flood debris, 64-000241; 2 males, 8 km W by N of Bald Hill, McIlwraith Range, Qld, 13°45'S, 143°22'E ±2 km, 500 m, 27 June - 12 July 1989, T.A. Weir, ANIC berlesate 1117, upper Leo Creek site, closed forest, leaf litter, 64-000243.

##### Other material.

**ANIC:** 1 male, Noah Creek, Qld, 16°07'S, 145°25'E ±2 km, 50 m, 21 June 1971, R.W. Taylor and J. Feehan, ANIC berlesate 321, rainforest, 64-000245; 1 female, 1 stadium 7 female, Cooper Creek near Daintree, Qld, 16°11'S, 145°26'E ±2 km, 50 m, 22 June 1971, R.W. Taylor and J. Feehan, ANIC berlesate 334, rainforest, 64-000246; 2 males, 1 female, Moses Creek, 4 km N by E of Mt Finnigan, Qld, 15°47'S, 145°17'E ±2 km, 14-16 October 1980, T. Weir, ANIC berlesate 696, 64-000244. **QM:** 2 males, 1 female, 1 stadium 7 male, 2.5 km SW of Mt Hartley via Cooktown, Qld, 15°47'45"S, 145°18'17"E ±500 m, 610 m, 24 April 1982, G. Monteith, D. Yeates and D. Cook, QM berlesate 400, rainforest, sieved litter, S92793; 2 females, Table Mountain, 10 km S of Cape Tribulation, Qld, 16°09'30"S, 145°26'07"E ±500 m, 320 m, 24 April 1983, G. Monteith and D. Cook, QM berlesate 542, rainforest, sieved litter, S92801; 1 male, 1 female, 3 stadium 7 males, Mt Finnigan summit, Qld, 15°49'06"S, 145°16'45"E ±500 m, 1100 m, 30 November 1985, G. Monteith and D. Cook, QM berlesate 700, rainforest, sieved litter, S92794; 1 male, 2 females, 1 stadium 6 female, same details but 21 November 1998, G. Monteith, QM berlesate 981, S92795; 1 stadium 7 female, Mt Misery summit via Shiptons Flat, Qld, 15°52'46"S, 145°13'32"E ±500 m, 850 m, 6 December 1990, G. Monteith, G. Thompson, R. Sheridan, D. Cook and L. Roberts, S92798; 1 female, Mt Pieter Botte, Qld, 16°04'37"S, 145°24'22"E ±500 m, 950 m, 21 November 1993, G. Monteith, H. Janetzki, L. Roberts and D. Cook, S92800; 1 male, 1 female, Mt Boolbun South, Qld, 15°57'10"S, 145°08'23"E ±500 m, 850-1000 m, 4-6 November 1995, G. Monteith, D. Cook and L. Roberts, S92796; 1 male, 1 stadium 7 male, same details but 850 m, 6 November 1995, G. Monteith, QM berlesate 896, rainforest, sieved litter, S92799; 1 female, 1 stadium 7 female, same details but QM berlesate 897, S92797.

##### Diagnosis.

Readily distinguished from *Notopyrgodesmus lanosus* sp. n. in having ozopores on porosteles on rings 5, 7, 9, 10, 12, 13, 15, 16, and in lacking ‘spines’ (tight clusters of hair-like cuticular outgrowths) on the tips of the paramedian tubercles. Readily distinguished from *Notopyrgodesmus weiri* sp. n. in larger size (adults 9-10 mm in length vs 6 mm) and in the lack of a deep notch separating the lateral marginal lobes on the paranota.

##### Description.

Colour in alcohol pale yellow, with brown encrusted soil particles on vertex, frons, collum, tergites, metatergites, preanal ring, gonocoxae and midline of sternites (Fig. 10A). Males and females with head + 20 rings. Male/female approximate measurements: length 9/10 mm; ring 12: overall width 1.8/1.8 mm, overall width/prozonite width 2.0/1.8, maximum vertical diameter 1.0/1.2 mm.

Head with vertex and frons roughened, clypeus smooth and setose. Postantennal groove deep; antennal sockets separated by ca 1× a socket diameter; antennomere relative widths 5>(3,4,6)>2, relative lengths 5>(3,4,5)>6.

Collum completely covering head in dorsal view (Fig. 3C); anterior margin of collum parallel to ground, weakly divided into 10 rounded lobes; main part of collum nearly perpendicular to ground in lateral view. Overall ring widths 2-17 about equal; rings 18 and 19 narrower.

Midbody metatergite sculpture sometimes obscured by encrusted soil particles, but clearly patterned in 2 transverse rows (Fig. 10A, B): 2 low paramedian tubercles; dorsum between paramedian tubercles with 2 anterior raised areas and triangular posterior raised area, apex of the latter pointed anteriorly; 2 (sometimes 3) low dorsolateral tubercles, the posterior tubercle largest, sometimes extending slightly beyond posterior margin of metatergite and set slightly more dorsal than anterior dorsolateral tubercle. A prominent, posterolaterally directed tubercle on posterior metatergal margin, just above paranotal base. Metatergite surface broken into complex but more or less regular mosaics of low raised areas between paramedian tubercles and dorsolateral tubercles, and between dorsolateral tubercles and posterior paranotal lobe. Posterior paramedian tubercles enlarged as apically rounded cones and directed posterodorsally on rings 17-19; ring 18 tubercles largest (Fig. 4D).

Paranota (Fig. 5D) arising low on body, very slightly directed posteriorly, declined at ca 30°, pitched slightly so anterior margin is lower than posterior margin. Ring 2 paranotum expanded anterodistally, lateral margin very weakly divided into 3 rounded lobes. From ring 3 onwards, anterior margin straight, undivided; 2 lateral marginal lobes (ring 3 sometimes undivided); posterior margin with deep notch basally. Rings 18 and 19 paranota directed posterolaterally.

Metatergal surface details obscured by soil particles and secretions, but some margins with very small, hair-like cuticular outgrowths with expanded tips; no setae evident on collum, tergites or metatergites. Prozonites with lozenge-shaped raised elements anterior to suture and subspherical knobs posterior to suture; suture marked by a ridge of longitudinally elongated elements. Limbus a single row of very thin lobes with more or less straight distal margins, and with 1–2 very thin spikes above each lobe (Fig. 2E).

Ozopores (Fig. 5D, 7F) on porosteles near posterolateral corner of paranota on rings 5, 7, 9, 10, 12, 13, 15, 16; not evident on 17–19; porosteles directed somewhat posterolaterally, cylindrical, apically slightly expanded.

Sternites slightly roughened, not setose, longer than wide; transverse impression slightly deeper than longitudinal. Legs short, hidden by paranota in dorsal view; relative podomere lengths (femur, tarsus)>prefemur>(postfemur, tibia). Spiracles not evident.

Preanal ring with 3+3 lobes; epiproct short, bluntly rounded, spinnerets in square array below apex; hypoproct rounded-triangular.

Gonopore a small, inconspicuous opening on medial surface of leg 2 coxa. Aperture transversely ovoid, ca 1/3 width of ring 7 prozonite. Gonocoxa roughly quadrangular in medial view; ventrolateral surface roughened and convex; medial surface forming gonocoel, this surface basally setose. Telopodite (Figs 8C, D) attached at anterodorsal corner of gonocoel, directed towards posteroventral corner. Telopodite without setae, cylindrical at base, distal two-thirds shaped like curled leaf, concave medially; apex acuminate; posterobasal margin curled medially; anterobasal margin similarly curved, extended posteriorly as subcylindrical solenomere bent slightly basally and terminating below apex of telopodite, solenomere apex narrowing but blunt. Prostatic groove following the curled posterobasal margin of telopodite to solenomere base, opening at solenomere tip. When retracted, gonocoxae forming two low, rounded mounds, their flat medial surfaces appressed with narrow longitudinal slit between, telopodites not visible.

Female with epigyne ca 1/3 ring width, slightly raised, rounded-rectangular with straight distal margin; cyphopods not examined.

##### Distribution.

**Figure 11.**
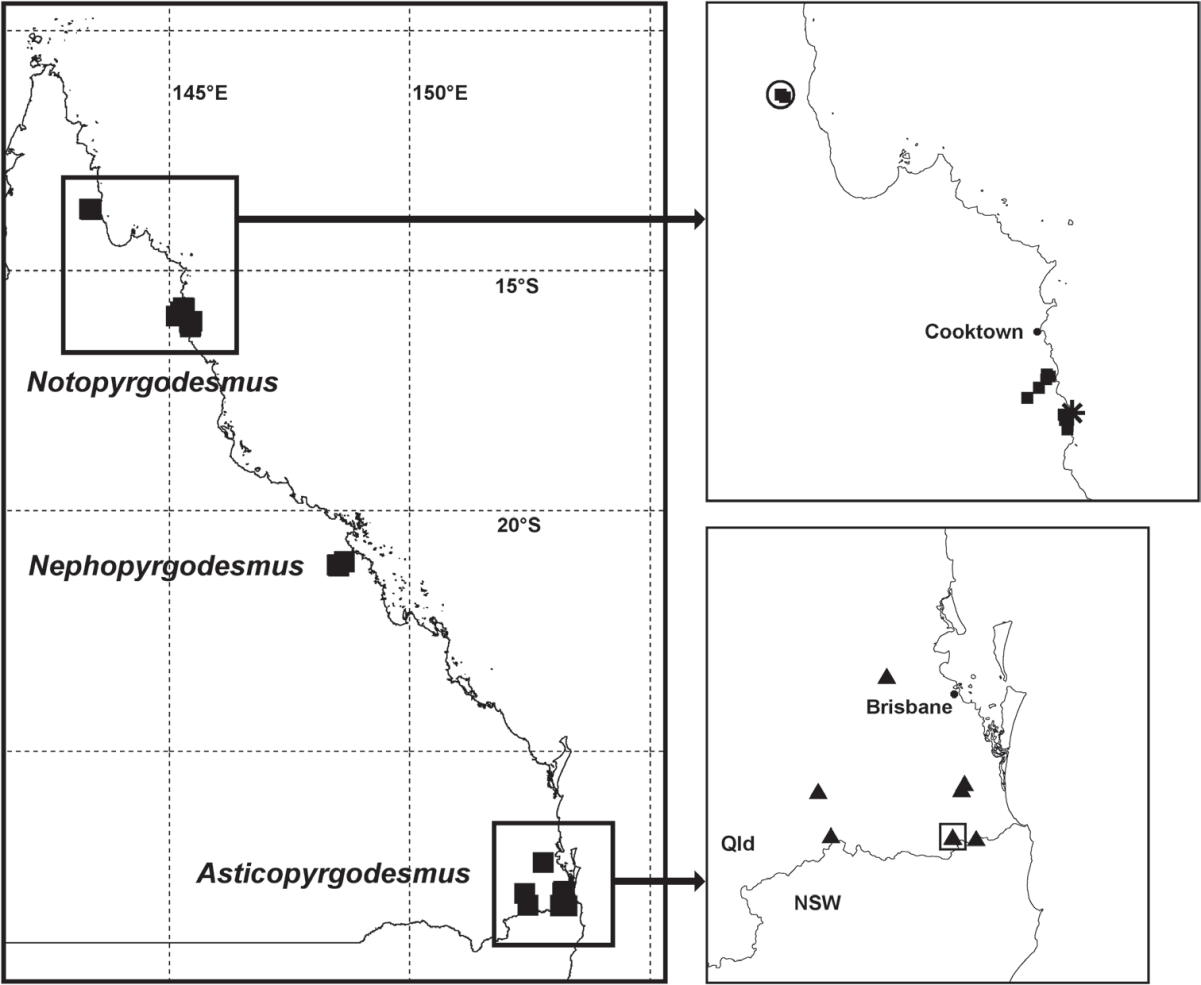
Localities in Queensland for Pyrgodesmidae described in this paper; see Fig. 1 for location within Australia. Northern Queensland map (top right): *Notopyrgodesmus kulla* sp. n. (filled squares), *Notopyrgodesmus lanosus* sp. n. (star) and *Notopyrgodesmus weiri* sp. n. (open circle). Southern Queensland map (bottom right): *Asticopyrgodesmus maiala* sp. n. (filled triangles), *Asticopyrgodesmus lamingtonensis* sp. n. (open square). Geographic projection; see text or Appendix for details of records. Qld = Queensland, NSW = New South Wales.

Wet forest on Cape York Peninsula south to the Daintree area in tropical northern Queensland, a north-south distance of ca 350 km (Fig. 11). Co-occurs with *Notopyrgodesmus weiri* sp. n. in the McIlwraith Range.

##### Etymology.

Acronym, noun in apposition, for the four Aboriginal clans who are traditional owners of the McIlwraith Range: Kaanju, Umpila, Lama Lama and Ayapathu (http://www.derm.qld.gov.au/parks/kulla-mcilwraith-range/culture.html; accessed 12 July 2012). KULLA is also the name of the national park located in the Range.

##### Remarks.

The specimens from the southern part of the *Notopyrgodesmus kulla* range (Fig. 10A) are mostly smaller (males ca 8 mm long, midbody overall width ca 1.5 mm) than those from the type locality and are less encrusted with soil particles.

#### Notopyrgodesmus lanosus

Mesibov sp. n.

urn:lsid:zoobank.org:act:D2D1FC34-764B-4695-8E7A-A4DD093270EF

http://species-id.net/wiki/Notopyrgodesmus_lanosus

Figs 3D , 4E , 5E , 6C, 6D , 10C

##### Holotype.

Male, Cape Tribulation area, Qld, 16°03' to 16°05'S, 145°28'E ±5 km, 21-28 March 1984, A. Calder and T. Weir, ANIC berlesate 943, rainforest on steep slopes, ANIC 64-000248 (in 2 pieces).

##### Paratypes.

**ANIC:** 1 male, 1 female, 1 stadium 7 male, 1 stadium 7 female, details as for holotype, 64-000247.

##### Other material.

None.

##### Diagnostic description.

As for *Notopyrgodesmus kulla* sp. n., differing in the following details (see also Figs 3D, 5E, 10C):

Male ca 7 mm long; ring 12: overall width 1.3 mm, overall width/prozonite width 1.9, maximum vertical diameter 0.8 mm; female very slightly larger. Anterior paramedian tubercles conical, apically rounded; posterior paramedian tubercles equally tall, apically with 2 short, rounded cones (Fig. 4E). In male, female and stadium 7 juveniles, the collum, tergites, metatergites, paranota, preanal ring and epiproct irregularly and densely covered with long, hair-like structures with expanded tips (Figs 6C, D); these structures tightly clustered at each of the 3 paramedian tubercle tips, the cluster appearing as a single ‘spine’ at low magnification. Porosteles on rings 5, 7, 9, 12, 15 in male, female, stadium 7 juveniles. Gonopods not significantly different in form from those of *Notopyrgodesmus kulla* sp. n., although proportionately smaller.

##### Distribution.

So far known only from rainforest at the type locality in tropical northern Queensland (Fig. 11).

##### Etymology.

Latin *lanosus* (‘woolly’), referring to the dense covering of fine, hair-like structures in this species; adjective.

#### Notopyrgodesmus weiri

Mesibov sp. n.

urn:lsid:zoobank.org:pub:3804BB73-6460-434F-91C8-225173EF2FDA

http://species-id.net/wiki/Notopyrgodesmus_weiri

Figs 2B , 3E, 3F , 4F , 5F , 6B , 10D

##### Holotype.

Male, 11 km W by N of Bald Hill, McIlwraith Range, Qld, 13°44'S, 143°20'E ±2 km, 520 m, 27 June - 12 July 1989, T.A. Weir, ANIC berlesate 1112, search party campsite, closed forest, flood debris, ANIC 64-000250.

##### Paratypes.

**ANIC:** 1 male, details as for holotype 64-000251; 1 male, same data but ANIC berlesate 1114, leaf litter and flood debris, 64-000249.

##### Other material.

None.

##### Diagnostic description.

As for *Notopyrgodesmus kulla* sp. n., differing in the following details (see also Figs 2B, 3E, 3F, 6B):

Male ca 6 mm long; ring 12: overall width 1.3 mm, overall width/prozonite width 2.4, maximum vertical diameter 0.6 mm. Paranota (Fig. 5F, 10D) divided laterally into anterior and posterior lobes by prominent notch; porostele-bearing posterior lobes distinctly shorter than anterior lobes on same ring. Paramedian tubercles conical, apically rounded, beginning ring 14 increasingly extended, the posterior tubercle taller and directed posterodorsally (Fig. 4F). No prominent tubercle on posterior metatergal margin above base of paranotum. Gonopods not significantly different in form from those of *Notopyrgodesmus kulla* sp. n., although proportionately smaller.

##### Distribution.

So far known only from wet forest at the type locality on Cape York Peninsula, Queensland (Fig. 11). Co-occurs with *Notopyrgodesmus kulla* sp. n.

##### Etymology.

To honour Tom Weir, CSIRO entomologist and diligent collector; adjective.

##### Remarks.

Since *Notopyrgodesmus kulla* sp. n. and *Notopyrgodesmus weiri* co-occur, it is clear that mate recognition in these species depends in syntopy on factors, including body size, other than gonopod form.

## Discussion

The prozonite sculpture found in the three new Australian genera (Figs 2F–H) is very similar to that illustrated by Akkari and Enghoff (2011b) for one species each in the pyrgodesmid genera *Cryptocorypha* Attems, 1907 and *Cynedesmus* Cook, 1895, namely irregular lozenge-shaped projections ahead of the suture and rounded, dome-like projections behind the suture, with the suture marked by a ridge of longitudinally extended projections. The new genera also have the lobe-and-spike style of limbus illustrated by Akkari and Enghoff (2011b) for a species of *Cynedesmus*, although in the Australian genera the number of spikes above a lobe on the lower part of the limbus can vary from one to two (e.g., Fig. 2C) on a single ring of a single individual. I have not checked the mandibles of any of the Australian species for the presence of a molar hook, a structure which may be diagnostic for Pyrgodesmidae (Akkari and Enghoff 2011a).

The diagnoses I give above for the three new Australian genera are only intended to distinguish one from the other, and will need to be substantially revised and expanded when the many other native Australian Pyrgodesmidae are described. The diagnoses are not intended for use in distinguishing Australian from non-Australian genera. A diagnosis of that kind would require more information on reliable generic characters and character states than is currently available for this millipede family, whose systematics is confused:

*With more than 170 described, mostly monobasic genera, the Pyrgodesmidae is a family in dire need of revisionary work. This is mainly due to the policy of relying mostly on conspicuous somatic characters for the definition of genera rather than on perhaps more reliable characters, such as gonopodal structures, which are more complex and comparatively more difficult to describe... Many other contemporary authors have commented on the lamentable state of pyrgodesmid taxonomy*...(Akkari and Enghoff 2011a, p. 61)

I also prefer not to speculate on evolutionary affinities by linking the new Australian genera with existing genera on the basis of shared character states which are unlikely to be genus-level synapomorphies, such as the collum covering/not covering the head in dorsal view and presence/absence of porosteles, or with character states known to vary at the species level (e.g. porostele formula in *Notopyrgodesmus*).

The new Australian genera, however, are clearly distinct from the four pyrgodesmid genera previously described from closely neighbouring landmasses, namely *Pixodesmus* Carl, 1926 and *Plethodesmus* Carl, 1926 from New Caledonia, and *Evurodesmus* Silvestri, 1920 and *Lobiferodesmus* Silvestri, 1920 from Papua New Guinea. None of the new Australian species has the double transverse row of very large tubercles on the collum in *Pixodesmus*, the three clearly separated paramedian tubercles on posterior rings seen in *Plethodesmus*, or the combination of three transverse rows of metatergal tubercles and presence of porosteles in *Evurodesmus*. *Lobiferodesmus* and *Asticopyrgodesmus* are alike in having no paramedian tubercles and a collum not covering the head, but species of the former have short, stout gonopod telopodites, while the telopodites in the two *Asticopyrgodesmus* species are comparatively long and slender, although bent. The gonopod telopodites of the four other Australian species are also distinct from those described and illustrated for *Pixodesmus*, *Plethodesmus* and *Evurodesmus*,none of which have the flagellum-like solenomere of *Nephopyrgodesmus eungella*, or the leaf-like telopodite tip of the *Notopyrgodesmus* species.

Whether any of the native Australian pyrgodesmids can be accommodated in genera known from elsewhere in the world is a question which depends for its answer on the much-needed family-level revision.

## Supplementary Material

XML Treatment for Asticopyrgodesmus

XML Treatment for Asticopyrgodesmus maiala

XML Treatment for Asticopyrgodesmus lamingtonensis

XML Treatment for Nephopyrgodesmus

XML Treatment for Nephopyrgodesmus eungella

XML Treatment for Notopyrgodesmus

XML Treatment for Notopyrgodesmus kulla

XML Treatment for Notopyrgodesmus lanosus

XML Treatment for Notopyrgodesmus weiri

## Appendix

Specimen records of Australian Pyrgodesmidae. (doi: 10.3897/zookeys.217.3809.app) File format: Comma-separated values files (CSV).

**Explanation note:** Specimen records of Australian Pyrgodesmidae have been compiled as separate CSV files for named and unidentified species. Both files are available on the ZooKeys website in a single zipped file.
